# Effects of the cyclooxygenase-2 inhibitor nimesulide on cerebral infarction and neurological deficits induced by permanent middle cerebral artery occlusion in the rat

**DOI:** 10.1186/1742-2094-2-3

**Published:** 2005-01-18

**Authors:** Eduardo Candelario-Jalil, Noël H Mhadu, Armando González-Falcón, Michel García-Cabrera, Eduardo Muñoz, Olga Sonia León, Bernd L Fiebich

**Affiliations:** 1 Department of Pharmacology, University of Havana (CIEB-IFAL), Havana 10600, Cuba; 2 Neurochemistry Research Group, Department of Psychiatry, University of Freiburg Medical School, Hauptstrasse 5, D-79104 Freiburg, Germany; 3 Departamento de Biología Celular, Fisiología e Inmunología. Universidad de Córdoba, Avda Menéndez Pidal s/n. 14004, Córdoba, Spain; 4 VivaCell Biotechnology GmbH, Ferdinand-Porsche-Str. 5, D-79211 Denzlingen, Germany

## Abstract

**Background:**

Previous studies suggest that the cyclooxygenase-2 (COX-2) inhibitor nimesulide has a remarkable protective effect against different types of brain injury including ischemia. Since there are no reports on the effects of nimesulide on permanent ischemic stroke and because most cases of human stroke are caused by permanent occlusion of cerebral arteries, the present study was conducted to assess the neuroprotective efficacy of nimesulide on the cerebral infarction and neurological deficits induced by permanent middle cerebral artery occlusion (pMCAO) in the rat.

**Methods:**

Ischemia was induced by permanent occlusion of the middle cerebral artery in rats, *via *surgical insertion of a nylon filament into the internal carotid artery. Infarct volumes (cortical, subcortical and total) and functional recovery, assessed by neurological score evaluation and rotarod performance test, were performed 24 h after pMCAO. In initial experiments, different doses of nimesulide (3, 6 and 12 mg/kg; i.p) or vehicle were administered 30 min before pMCAO and again at 6, 12 and 18 h after stroke. In later experiments we investigated the therapeutic time window of protection of nimesulide by delaying its first administration 0.5–4 h after the ischemic insult.

**Results:**

Repeated treatments with nimesulide dose-dependently reduced cortical, subcortical and total infarct volumes as well as the neurological deficits and motor impairment resulting from permanent ischemic stroke, but only the administration of the highest dose (12 mg/kg) was able to significantly (P < 0.01) diminish infarct volume. The lower doses failed to significantly reduce infarction but showed a beneficial effect on neurological function. Nimesulide (12 mg/kg) not only reduced infarct volume but also enhanced functional recovery when the first treatment was given up to 2 h after stroke.

**Conclusions:**

These data show that nimesulide protects against permanent focal cerebral ischemia, even with a 2 h post-treatment delay. These findings have important implications for the therapeutic potential of using COX-2 inhibitors in the treatment of stroke.

## Background

The brain is highly sensitive to disturbance of its blood supply. Stroke is a devastating disease and is the third most common cause of death, and the most common cause of motor and mental disability in adults, in developing countries [1]. Complex pathophysiological events occur in brain during ischemic processes, and these are considered responsible for cell damage leading to neuronal death (for review see [2,3]). However, it is now generally accepted that the mammalian brain may be more resistant to ischemia than previously thought. This raises the possibility of therapeutic intervention before brain damage has become irreversible.

A number of interacting and sequentially evoked events tend to reinforce the initial ischemic insult. A key role in these processes is played by post-ischemic inflammation. The Ca^2+^-related activation of intracellular second messenger systems, the increase in reactive oxygen species formation, as well as hypoxia itself triggers the expression of a large number of pro-inflammatory genes following cerebral ischemia. Thus, mediators of inflammation such as platelet-activating factor (PAF), tumor necrosis factor α (TNFα), interleukin 1β (IL-1β), chemokines (IL-8, monocyte chemoattractant protein-1) and other pro-inflammatory factors are produced by the ischemic brain tissue [3]. In addition, the expression of adhesion molecules with the subsequent infiltration of polymorphonuclear leukocytes occurs following ischemic stroke. Results from several studies also suggest that the marked and sustained expression of inflammation-related enzymes such as inducible nitric oxide synthase (iNOS) and cyclooxygenase-2 (COX-2) plays an important role in the secondary events that amplify cerebral injury after ischemia [4-12].

Nimesulide (N-(4-nitro-2-phenoxyphenyl)-methanesulfonamide) is a non-steroidal anti-inflammatory drug with potent effects. It shows a high affinity and selectivity for COX-2 with a COX-2/COX-1 IC_50 _selectivity ratio of 0.06 (whole blood assay) [13]. Nimesulide readily crosses the intact blood-brain barrier in both humans and rodents [13,14]. Several recent studies have demonstrated a marked neuroprotective effect of nimesulide on chronic cerebral hypoperfusion [15], kainate-induced excitotoxicity [16], quisqualic acid-induced neurodegeneration [17], diffuse traumatic brain injury [18,19], glutamate-mediated apoptotic damage [20] and induction of the expression of the B subunit of endogenous complement component C1q (C1qB) in transgenic mice with neuronal overexpression of human COX-2 [21].

Recently, we have found a significant neuroprotective effect of nimesulide both in global cerebral ischemia [10,22], a type of injury that mimics the clinical situation of cardio-respiratory arrest, and in a rat model of ischemic stroke induced by the transient (1 h) occlusion of the middle cerebral artery [12].

Since most cases of human ischemic stroke are caused by permanent occlusion of cerebral arteries [23-26], the present study was conducted to assess whether nimesulide would also show neuroprotective efficacy on the cerebral infarction induced by permanent middle cerebral artery occlusion (pMCAO) in the rat, a clinically relevant model of ischemic stroke. The effects of the COX-2 inhibitor nimesulide had not been previously investigated in a model of permanent ischemic stroke.

## Methods

### Animals

Male Sprague-Dawley rats (CENPALAB, Havana, Cuba) weighing 280–340 g at the time of surgery were used in the present study. Our institutional animal care and use committee approved the experimental protocol (No. 02/67). The animals were quarantined for at least 7 days before the experiment. Animals were housed in groups in a room whose environment was maintained at 21–25°C, 45–50 % humidity and 12-h light/dark cycle. They had free access to pellet chow and water. Animal housing, care, and application of experimental procedures were in accordance with institutional guidelines under approved protocols.

### Induction of permanent focal cerebral ischemia in the rat

Rats were anesthetized with chloral hydrate (300 mg/kg body weight, i.p.). Once surgical levels of anesthesia were attained (assessed by absence of hind leg withdrawal to pinch), ischemia was induced by using an occluding intraluminal suture as described previously [27-29]. Briefly, the right common carotid artery (CCA) was exposed by a ventral midline neck incision and ligated with a 3-0 silk suture. The pterygopalatine branch of the internal carotid artery was clipped to prevent incorrect insertion of the occluder filament. Arteriotomy was performed in the CCA approximately 3 mm proximal to the bifurcation and a 3-0 monofilament nylon suture, whose tip had been rounded by being heated near a flame was introduced into the internal carotid artery (ICA) until a mild resistance was felt (18–19 mm). Mild resistance to this advancement indicated that the intraluminal occluder had entered the anterior cerebral artery and occluded the origin of the anterior cerebral artery, the middle cerebral artery (MCA) and posterior communicating arteries [27]. After the advancement of the nylon suture, the ICA was firmly ligated with a 3-0 silk suture. The incision was closed and the occluding suture was left in place until sacrificing the animals. The duration of surgery did not exceed 12 min in any case. The animals were allowed to recover from anesthesia and to eat and drink freely. The body temperature was strictly controlled during and after ischemia. To allow for better postoperative recovery, we chose not to monitor physiological parameters in the present study because additional surgical procedures are needed for this monitoring. Nevertheless, we performed separate experiments to investigate the effects of nimesulide on major physiological variables such as mean arterial blood pressure, blood glucose, rectal temperature, hematocrit, blood pH and blood gases (pO_2 _and pCO_2_). The effects observed with nimesulide in the present study were not related to modification of physiological variables since these parameters did not differ between nimesulide-treated and vehicle-treated rats (data not shown). These findings are in agreement with our previous results [10,12], suggesting that nimesulide does not significantly change major physiological variables.

### Neurological evaluation

An unaware independent observer performed the neurological evaluations prior to the sacrifice of the animals according to a six-point scale: 0= no neurological deficits, 1= failure to extend left forepaw fully, 2= circling to the left, 3= falling to left, 4= no spontaneous walking with a depressed level of consciousness, 5= death [30,31].

### Assessment of functional outcome

Motor impairment in this study was assessed with the use of the accelerating rotarod (Ugo Basile, Varese, Italy, Model 7750). Rats were given 2 training sessions 10 minutes apart before surgery. Rats were first habituated to the stationary rod. After habituation they were exposed to the rotating rod. The rod was started at 2 rpm and accelerated linearly to 20 rpm within 300 sec. Latency to fall off the rotarod was then determined before ischemia (presurgery) and before sacrificing the animals. Animals were required to stay on the accelerating rod for a minimum of 30 sec. If they were unable to reach this criterion, the trial was repeated for a maximum of five times. The two best (largest) fall latency values a rat could achieve then were averaged and used for data analysis. Rats not falling off within 5 min were given a maximum score of 300 seconds [32,33]. A sham-operated group was also included (n = 8). The investigator performing the rotarod test did not know the identity of the experimental groups until completion of data analysis.

### Quantification of brain infarct volume

The method for quantification of infarct volume was performed exactly as reported by others [34,35]. Briefly, the animals were sacrificed under deep anesthesia and brains were removed, frozen, and coronally sectioned into six 2-mm-thick slices (from rostral to caudal, first to sixth). The brain slices were incubated for 30 min in a 2% solution of 2,3,5-triphenyltetrazolium chloride (TTC) (Sigma Chemical Co.) at 37°C and fixed by immersion in a 10% phosphate-buffered formalin solution. Six TTC-stained brain sections per animal were placed directly on the scanning screen of a color flatbed scanner (Hewlett Packard HP Scanjet 5370 C) within 7 days. Following image acquisition, the image were analyzed blindly using a commercial image processing software program (Photoshop, version 7.0, Adobe Systems; Mountain View, CA). Measurements were made by manually outlining the margins of infarcted areas. The unstained area of the fixed brain section was defined as infarcted. Cortical and subcortical uncorrected infarcted areas and total hemispheric areas were calculated separately for each coronal slice. Total cortical and subcortical uncorrected infarct volumes were calculated by multiplying the infarcted area by the slice thickness and summing the volume of the six slices. A corrected infarct volume was calculated to compensate for the effect of brain edema. An edema index was calculated by dividing the total volume of the hemisphere ipsilateral to pMCAO by the total volume of the contralateral hemisphere. The actual infarct volume adjusted for edema was calculated by dividing the infarct volume by the edema index [36-38]. Infarct volumes are expressed as a percentage of the contralateral (control) hemisphere. The investigators who performed the image analysis were blinded to the study groups.

## Experimental design

### Time course of lesion development after pMCAO

At various times after pMCAO (4, 8, 12, 24 and 48 h, n = 6–8 per group) the animals were sacrificed and the brains were quickly removed, sectioned and stained as previously described in order to calculate the infarct volume.

### Evaluation of nimesulide's effects: dose-response experiment

In order to evaluate the effect of nimesulide administration on rat focal cerebral ischemia, three different doses of nimesulide (3, 6 and 12 mg/kg) were given to rats by intraperitoneal administration 30 min before the onset of pMCAO (n = 7–9 animals per group). Additional doses were given at 6, 12 and 18 h after stroke. This treatment schedule and dosage range was based on the pharmacokinetic profile of nimesulide [39] and on our previous experience with this compound in models of cerebral ischemia [10,12]. We also studied the effect of a single dose of nimesulide (12 mg/kg; i.p.) given 30 min before ischemia (n = 8). A single injection vehicle-treated group was also included (n = 7).

### Assessment of the therapeutic time window for the neuroprotective effect of nimesulide in pMCAO

After investigating the dose-response relationship, we studied the effect of nimesulide (12 mg/kg; i.p.) when administered 0.5, 1, 2, 3 or 4 h after ischemia (n = 8–11 animals per group). The corresponding vehicle-treated groups were included as controls (n = 7–10 rats per group). Three additional doses were given every 6 h after the first treatment with nimesulide or vehicle exactly as described before for the repeated treatment schedule in the dose-response experiment. After completing the neurological evaluation and rotarod performance at 24 h after permanent focal cerebral ischemia, animals were sacrificed and the brains were removed to calculate the infarct size.

### Data analysis

Data are presented as means ± S.D. Values were compared using t-test (two groups) or one-way ANOVA with *post-hoc *Student-Newman-Keuls test (multiple comparison). Neurological deficit scores were analyzed by Kruskal-Wallis non-parametric ANOVA followed by the Dunn test (multiple comparison) or Mann-Whitney test for analysis of individual differences. Rotarod performance was expressed as a percentage of pre-surgery values for each rat and analyzed by ANOVA for repeated measures followed by the Student-Newman-Keuls test. Differences were considered significant when p < 0.05.

## Results

### Time course of the development of cerebral infarction and neurological deficits after pMCAO

**Figure 1:**
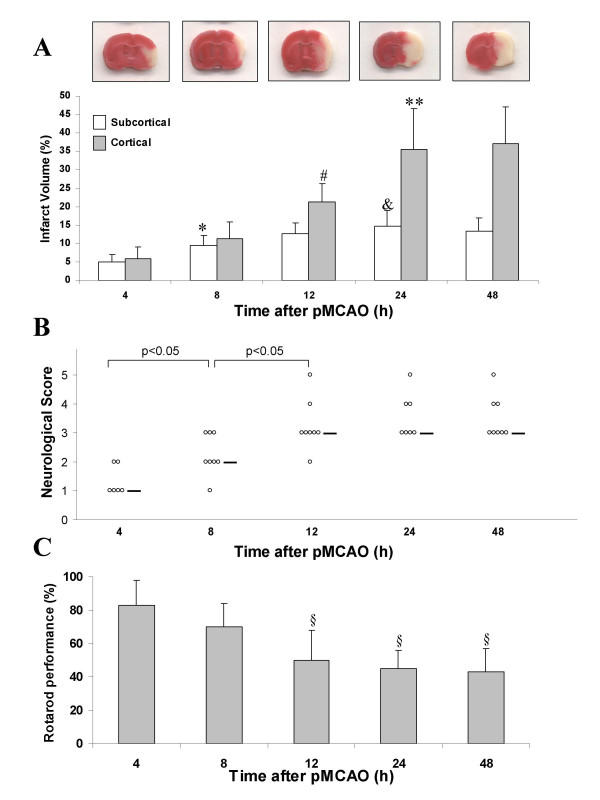
Temporal development of focal cerebral infarction induced by permanent middle cerebral artery occlusion (pMCAO). (**A**): Evolution of cortical and subcortical infarct volumes after pMCAO in rats. Representative TTC-stained sections at different times after stroke are shown in the insets. (**B**) and (**C**): Time course of the increase of neurological deficits and motor impairment induced by pMCAO. Infarct volumes are expressed as a percentage of the contralateral (control) hemisphere. Bars represent the group mean ± SD. * p < 0.05 with respect to subcortical infarct volume at 4 h. ^&^p < 0.05 with respect to subcortical infarct volume at 8 h. ^# ^p < 0.05 with respect to cortical infarct volume at 8 h. ** p < 0.05 with respect to cortical infarct volume at 12 h. ^§ ^p < 0.05 with respect to 4 and 8 h. The horizontal bar in Panel B shows the median neurological score.

The temporal evolution of the lesion volumes is presented in Fig. 1A as the cortical and subcortical components of the infarction. Subcortical injury was evident in TTC-stained coronal sections as early as 4 h after permanent stroke (see insets of TTC-stained sections at different times after stroke in Fig. 1A). Subcortical lesion was maximal between 8 and 12 h after pMCAO, although there was a slight but significant increase between 8 and 24 h when the overall comparison was performed (one-way ANOVA, followed by Student-Newman-Keuls test). Nevertheless, the Student's t-test analysis failed to detect any significant increase between 12 and 24 or 48 h post-injury, thus indicating that the subcortical damage reached maximal values by 12 h after the insertion of the occluding filament (Fig. 1A). On the other hand, cortical damage progressed more slowly; it was detected at 4 h after pMCAO, and by 8 h there was an increase of the infarct but this was not statistically significant as compared to that at 4 h. On the contrary, there was a significant (p < 0.05) increase in the lesion when the infarction at 12 h is compared with that at 4 or 8 h, and a more dramatic increase of damage is seen at 24–48 h after stroke, a time at which the cortical infarct volume is maximal in this model as shown in Fig. 1A.

With regard to the neurological deficits and motor impairment induced by pMCAO (assessed by the neurological score and accelerating rotarod test), it is important to emphasize the fact that these parameters were maximal by 12 h after stroke and the animals did not show any further increase in the neurological deficits or motor impairment after 24 or 48 h of the occlusion, as depicted in Fig. 1B and Fig. 1C. Based on these results, we decided to evaluate the effects of nimesulide after 24 h of pMCAO.

### Effects of different doses of nimesulide on infarct volume and functional outcome after pMCAO

Repeated treatments with nimesulide dose-dependently reduced cortical, subcortical and total infarct volumes in the permanent model of stroke, although only the administration of the highest dose (12 mg/kg) was able to significantly (P < 0.01) diminish brain damage (Table 1). There was a trend towards a reduction in lesion volumes in animals treated with nimesulide 6 mg/kg, but this effect was not confirmed by the statistical analysis of the data. Unlike the long-term treatment paradigm, the administration of a single dose of nimesulide (12 mg/kg) 30 min before pMCAO failed to significantly reduce total infarct volume, though a modest neuroprotective effect was seen in the subcortical areas as shown in Table 1.

**Table 1:** Effect of different doses of the cyclooxygenase-2 inhibitor nimesulide on total, cortical and subcortical infarct volumes in a rat model of permanent focal cerebral ischemia.

**Treatment**	**Total infarct volume (%)**	**Cortical infarct volume (%)**	**Subcortical infarct volume (%)**
*Repeated doses*			
Vehicle (n = 9)	56.1 ± 11.4	41.6 ± 10.3	12.6 ± 4.5
Nimesulide 3 mg/kg (n = 7)	54.9 ± 14.9	38.1 ± 17.2	14.3 ± 2.4
Nimesulide 6 mg/kg (n = 8)	41.4 ± 12.3	31.8 ± 9.3	9.7 ± 3.5
Nimesulide 12 mg/kg (n = 9)	34.1 ± 13.8 **	24.6 ± 11.2 **	7.1 ± 3.9 *
*Single dose*			
Vehicle, single dose (n = 7)	55.2 ± 15.5	43.9 ± 10.9	13.2 ± 4.2
Nimesulide 12 mg/kg, single dose (n = 8)	49.5 ± 11.7	39.4 ± 11.8	9.1 ± 3.1^&^

Data are mean ± S.D. * P < 0.05 and ** P < 0.01 compared to vehicle. One-way ANOVA followed by Student-Newman-Keuls post-hoc test. ^&^P < 0.05 compared to vehicle single dose (Student's t-test).

Interestingly, repeated treatments with 6 and 12 mg/kg of nimesulide were similarly effective in reducing the neurological deficits and the motor impairment resulting from pMCAO (Table 2). This effect was not accompanied by a significant reduction in infarct volume in the case of the dose of 6 mg/kg (Table 1). No neuroprotective effect of nimesulide was observed on the neurological score or rotarod performance when this COX-2 inhibitor was administered as a single dose (12 mg/kg) before the onset of ischemia (Table 2).

**Table 2:** Effect of different doses of nimesulide on neurological deficits and functional outcome (evaluated using the rotarod test) following permanent middle cerebral artery occlusion in the rat.

**Treatment**	**Neurological Score**	**Rotarod performance (% of presurgery levels)**
Sham-operated control (n = 8)	0	128 ± 21
*Repeated doses*		
Vehicle (n = 9)	3 (3–5)	49 ± 18
Nimesulide 3 mg/kg (n = 7)	3 (2–4)	64 ± 13
Nimesulide 6 mg/kg (n = 8)	2 (1–5) *	89 ± 20 **
Nimesulide 12 mg/kg (n = 9)	2 (1–4) **	84 ± 14 **
*Single dose*		
Vehicle, single dose (n = 7)	3 (3–5)	43 ± 21
Nimesulide 12 mg/kg, single dose (n = 8)	3.5 (2–5)	52 ± 19

Values show the median and range (neurological score) and means ± S.D. (rotarod performance). For the analysis of neurological score data, the Kruskal-Wallis nonparametric ANOVA followed by Dunn test (multiple comparison) or Mann-Whitney test for analysis of individual differences were used. For the statistical analysis of rotarod performance results, ANOVA followed by Student-Newman-Keuls post-hoc test was employed. * P < 0.05 and ** P < 0.01 compared to vehicle.

### Therapeutic time window for nimesulide protection in rats subjected to pMCAO

**Figure 2:**
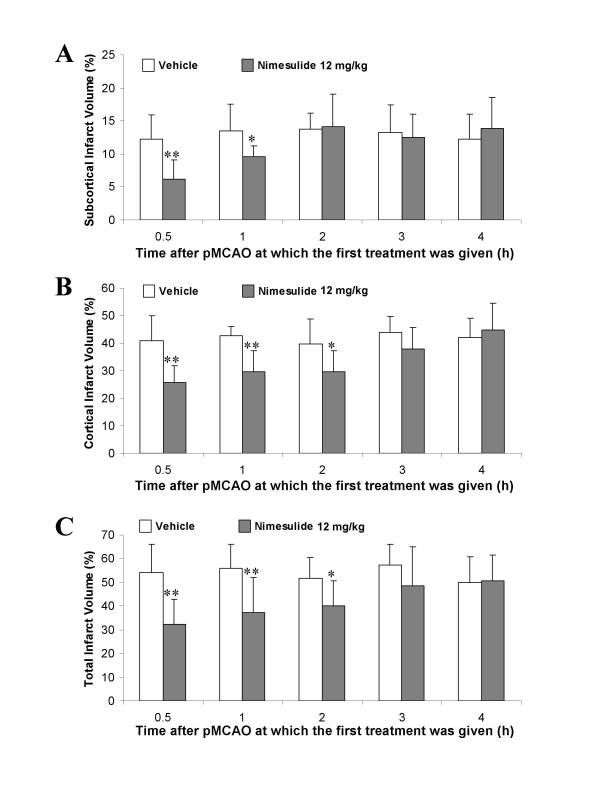
Reduction of subcortical (**A**), cortical (**B**) and total (**C**) infarct volumes by the cyclooxygenase-2 inhibitor nimesulide (12 mg/kg; i.p.) when its first administration was delayed for several hours after the onset of permanent stroke. Nimesulide reduced the infarct size in animals treated at 0.5 (n = 8), 1 (n = 9) and 2 h (n = 9), but not at 3 (n = 11) and 4 h (n = 9) after pMCAO, compared to vehicle-treated and time-comparable control groups (n = 7–9 per group). Infarct volumes are expressed as a percentage of the contralateral (control) hemisphere and the data are represented as the mean ± SD. * p < 0.05 and ** p < 0.01 with respect to vehicle (Student's t-test).

In this experiment we investigated the effect of nimesulide (12 mg/kg) in a situation in which its first administration was delayed for 0.5–4 h after the ischemic challenge. A significant reduction in subcortical infarct volume was observed when the treatment was delayed until 0.5–1 h after pMCAO, but this protective effect of nimesulide was not evident when administered after 2–4 h of the onset of permanent occlusion (Fig. 2A). In the case of cortical infarction, nimesulide diminished lesion volume when treatment was delayed until 2 h after the ischemic insult (Fig. 2B). Similar results were found for total infarct volume as shown in Fig. 2C, though as expected, an overall decline of the neuroprotective effect with post-treatment time was observed.

Of interest is the finding that nimesulide not only reduced infarct volume but also enhanced functional recovery when the first treatment is given 2 h after permanent ischemic stroke. Post-ischemic treatment with nimesulide significantly reduced neurological deficits and increased the fall latencies to remain on the accelerating rotarod as compared to those rats given only the vehicle (Table 3). However, this protective effect was lost when the first administration is delayed until 3–4 h after the occlusion of the middle cerebral artery as presented in Table 3.

**Table 3:** Effect of delayed administration of nimesulide (12 mg/kg; i.p.) on neurological deficit score and rotarod performance after permanent middle cerebral artery occlusion (pMCAO) in rats. Vehicle or nimesulide was administered 0.5, 1, 2, 3, or 4 h after stroke.

	**Neurological Score**		**Rotarod performance (%)**	
**Time after stroke (h)**	**Vehicle**	**Nimesulide**	**Vehicle**	**Nimesulide**

**0.5**	3 (2–5)	2 (1–3) **	44 ± 17	81 ± 18 **
**1**	3 (2–5)	2 (1–4) **	40 ± 21	85 ± 22 **
**2**	3 (3–5)	2 (1–5) *	50 ± 11	73 ± 13 *
**3**	3 (2–5)	3 (2–5)	47 ± 16	60 ± 15
**4**	3.5 (2–5)	3 (2–5)	52 ± 23	59 ± 14

Values represent the median and range (neurological score) and means ± S.D. (rotarod performance). *P < 0.05 and **P < 0.01 compared with the corresponding vehicle-treated group.

## Discussion

The present study was prompted by our previous encouraging results with nimesulide in a model of transient focal cerebral ischemia, which show that this COX-2 inhibitor is able to potently reduce infarct volume and improve functional recovery [12]. These neuroprotective effects are also observed when treatment is delayed until even 24 h after the onset of ischemia [12]. Since we believe that it is very important to perform thorough, multifactorial and well-designed pre-clinical studies before assuming definitive conclusions on the neuroprotective effect of any compound, and considering that in stroke patients a very early spontaneous recanalization of an obstructed brain vessel is, unfortunately, only rarely found, we conducted the present investigation to shed more light into the effects of nimesulide on ischemic damage using a permanent stroke model in the rat considering that this model might be more relevant to the clinical situation of stroke, as suggested previously [23-26].

The core findings of this study are: (i) administration of clinically relevant doses of nimesulide confers protection against the damage induced by permanent focal cerebral ischemia in two modalities (reduction of infarct size, and improvement of functional outcome) and (ii) nimesulide's neuroprotection is still evident when the first administration is delayed until 2 h after the onset of stroke.

Depending on the experimental conditions, the temporal evolution of ischemic damage may vary considerably [30,40,41]. Thus, it is very important to characterize the time course of brain damage, especially if one wants to interpret correctly the effects of a given compound using delayed treatment schedules. Our results showed that in the permanent model of stroke induced by the occlusion of the middle cerebral artery using an intraluminal suture, the infarct size progresses very fast in the subcortical areas (mainly striatum) and much slower in cortical areas, but in general the evolution of damage is relatively quick, reaching maximal values by 24 h after the insertion of the filament (Fig. 1A). These findings are in line with those published previously in this model of stroke [42,43]. Although infarct size continues to increase between 12 and 24–48 h of ischemia (Fig. 1A), the neurological deficits and motor impairment reached their maximum by 12 h, and the animals did not showed any further deterioration of their neurological functions (Fig. 1B and 1C). This might reflect the fact that unlike ischemic injury to many other tissues, the severity of disability is not predicted well by the amount of brain tissue lost. For example, damage to a small area in the medial temporal lobe may lead to severe disability, while damage to a greater volume elsewhere has little effect on function [2]. There is not always a direct correlation between the lesion size and the severity of neurological deficits as demonstrated before in animal models [29,44] and in stroke patients [45]. For that reason, it is essential to evaluate the neuroprotective effects of agents by combining both histological and functional measures. The present study offers a good example of this: even when the lowest doses of nimesulide did not reduce infarct volume in pMCAO (Table 1), one can not minimize the beneficial effects of these doses since a significant reduction in neurological deficits and an improvement of rotarod performance were observed (Table 2). Thus, further studies would be required to better characterize the effects of the lowest doses of nimesulide (3 and 6 mg/kg) in models of cerebral ischemia.

Repeated treatments with nimesulide afforded a more remarkable neuroprotection than the administration of a single dose given before the insult (Tables 1 and 2). These data show the importance of continuous long-term administration after ischemic damage in clinical trials to achieve the maximal beneficial effects of neuroprotection by nimesulide.

Unfortunately, a large number of promising neuroprotective compounds identified from preclinical experiments have failed in clinical trials in stroke patients [3,45-47]. Although several factors may contribute to these disappointing results, an important issue is the 'therapeutic time window of protection', defined as the time period after the onset of ischemia during which administration of treatment is effective [48,49]. Most of the agents that confer protection in experimental animal models of stroke when given before or a short period after cerebral ischemia have failed in clinical studies [45,50]. Thus, the assessment of the therapeutic time window of protection is of paramount importance in pursuing future therapies to treat stroke victims.

Therefore, our next experiments were conducted to evaluate the effects of nimesulide when administered in a delayed treatment schedule in order to establish the therapeutic time window of protection of this COX-2 inhibitor in pMCAO, thus increasing predictive outcome in the clinic. Interestingly, reduction in infarct size and neurological deficits and improvement of rotarod performance were still observed when nimesulide treatment was delayed until 2 h after ischemia (Fig. 2A, Table 3).

It is important to compare our present results in pMCAO with those previously obtained in transient ischemia [12]. In the model of transient focal ischemia, the time window of nimesulide's neuroprotection extends over a 24 h period [12], and in other models of cerebral ischemia, the time window of protection of nimesulide is similarly wide [10,22,51]. These results have been also obtained with other COX-2 inhibitors (e.g., NS-398, SC58125 and rofecoxib) in models of transient ischemic stroke [4,52] and global cerebral ischemia [53,54]. These studies suggest that although the protective effects of COX-2 inhibitors are more beneficial when administered early after the ischemic insult, COX-2 selective inhibitors show a wide therapeutic window for the prevention of neuronal death in both focal and global ischemia.

However, our present results suggest that in permanent stroke, COX-2 inhibition by nimesulide is not as protective as in transient models (39 % of infarct reduction with pre-treatment in pMCAO vs. 60 % lesion reduction in transient ischemia with immediate treatment) and the therapeutic time window is narrower as compared to temporary occlusion models (2 h in pMCAO vs. 24 h in transient ischemia) as the present results (Tables 1 and 3, Fig. 2) and our recent studies indicate [12]. The vascular inaccessibility of nimesulide into the ischemic/infarcted region could be a plausible explanation for these findings considering that, unlike transient ischemia, in permanent ischemic stroke the protective effects of any drug/agent depend, in large part, on the ability of the compound to reach the ischemic areas mainly through passive diffusion. Although these findings on nimesulide's effects on stroke tempted us to conclude that COX-2 selective inhibitors are less protective in permanent than in transient stroke models, these results should be interpreted with caution since a structurally similar COX-2 inhibitor (NS-398) reduced permanent stroke damage in mice when the treatment started 24 h after MCA occlusion [55] and the same COX-2 inhibitor also reduced lesion size when administered starting 6 h after pMCAO in another previous study [5]. Apparent discrepancies between our present results and these two reports [5,55] might be due to different methods to induce pMCAO (intraluminal vs. distal MCAO involving craniectomy), the specific COX-2 inhibitor used, treatment paradigm or animal species. This emphasizes the importance of conducting more preclinical studies with COX-2 selective inhibitors before these agents could be used in clinical trials in stroke patients. Another issue that needs urgent consideration in future studies with COX-2 inhibitors in cerebral ischemia is the effect of long-term treatment since anti-inflammatory interventions could interfere with nervous regeneration/plasticity and recovery as demonstrated in some types of neuronal injury [56,57].

## Conclusion

In summary, the present study has evaluated for the first time the neuroprotective effects of the COX-2 inhibitor nimesulide in permanent focal cerebral ischemia, showing beneficial effects on reduction of infarct volume and improvement of functional recovery. This ability of nimesulide to diminish permanent ischemic damage is observed even when the first treatment was delayed 2 h after the ischemic episode. Taken together, these results have important implications for the therapeutic potential of using the COX-2 selective inhibitor nimesulide in the treatment of cerebral ischemia.

## List of abbreviations used

COX-2, cyclooxygenase-2; pMCAO, permanent middle cerebral artery occlusion; MCA, middle cerebral artery; TTC, 2,3,5-triphenyltetrazolium chloride; ANOVA, analysis of variance

## Competing interests

The author(s) declare that they have no competing interests.

## Authors' contributions

ECJ carried out the surgical procedures to induce stroke, participated in the design of the study and in the statistical analysis, reviewed the data and drafted the manuscript. NHM performed the evaluation of neurological deficits and rotarod performance. AGF and MGC performed the calculation of the infarct volumes and participated in the statistical analysis of the data. OSL, EM and BLF participated in the design and coordination of the study, reviewed the data, provided consultation and helped to draft the manuscript. OSL and BLF share senior authorship. All authors read and approved the final manuscript.
